# European Green Deal: Hebel für internationale Klima- und Wirtschaftsallianzen

**DOI:** 10.1007/978-3-662-62195-0_4

**Published:** 2020-10-26

**Authors:** Mischa Bechberger, Yannick Thiele, Kirsten Neumann

**Affiliations:** Institut für Innovation und Technik, Berlin, Germany; Institut für Innovation und Technik, VDI/VDE Innovation + Technik GmbH, 10623 Berlin, Deutschland

## Abstract

Um eine Stabilisierung des Erdklimas zu erreichen, musste der Erdatmosphäre rund ein Viertel des in ihr enthaltenen Kohlendioxids, CO_2_, entzogen werden. Eine gigantische, aber nicht unmögliche Aufgabe, zu deren Losung es allerdings einer gemeinsamen internationalen Anstrengung aller Lander bedarf. Der European Green Deal bietet dabei eine nicht zu unterschätzende Gelegenheit für die Reaktivierung internationaler Klima-Diplomatie.

Die Verlegung des 25. Weltklimagipfels (COP) 2019 von Santiago de Chile nach Madrid und dessen ergebnisloser Ausgang verstärken die Kritik an der Notwendigkeit von Gipfel-Treffen in der internationalen Klima-Diplomatie. Überlegungen nach alternativen Formaten sind nötiger denn je und werden durch die seit der Corona-Krise zunehmenden Rufe nach einer Verlangsamung oder gar einem Aussetzen der nicht sehr verbindlichen Klimaschutzregelungen (Klimareporter 2020) verstärkt. Neue Impulse liefert die mit dem Amtsantritt der EU-Kommission Ende des Jahres 2019 initiierte und von der Mehrheit der EU-Mitgliedsstaaten gebilligte Klimaschutz-Roadmap in Form des European Green Deal. Dieser benennt klar innovative Ansätze der internationalen Klima-Diplomatie etwa durch entsprechende Kooperationen mit China und Afrika. Dass diese vor allem aus Klimaschutzgründen dringend geboten wären, wird daran deutlich, dass selbst die durch den European Green Deal angestrebte Klimaneutralität den Klimawandel nur verlangsamen kann. Somit stellt sich die Frage, wie dem Stillstand in der internationalen Klima-Diplomatie durch Governance-Ansätze in Form neuer strategischer Klimaallianzen begegnet werden kann. Hier bietet die aktuelle Corona-Krise die Chance, dem Klimaschutz im globalen Rahmen neue und beschleunigende Impulse zu geben, denn diese schärft als exponentiell wachsende kritische Lage, nach derzeitigem Stand nach den ersten Monaten, unser Bewusstsein für die ebenfalls exponentiell wachsende Klimakrise (Bals et al. 2020).

Exponentielle Krisenkurven verlangen demnach stets eine Doppelstrategie: Erstens muss die Kurve massiv abgeflacht werden, um das nicht zu Bewältigende zu vermeiden (Eindämmung) und Kipp-Punkte nicht zu überschreiten, die menschenwürdige Lösungen kaum noch möglich machen. Zweitens geht es darum, das Unvermeidbare in den Griff zu bekommen (Bekämpfung). Sobald es bei der Bekämpfung der Corona-Krise nicht mehr um die Stilllegung der Wirtschaft, sondern um ihre Ankurbelung gehe, müsse diese Strategie mit der Eindämmung der Klimakrise verzahnt werden (Bals et al. 2020:1).

In der aktuellen Lage bietet es sich an, die bereits aufgelegten und in Planung befindlichen Konjunkturpakete auf nationalstaatlicher und europäischer Ebene mit dem EU Green Deal intelligent zu verknüpfen. Dies forderte auch im Vorfeld des Petersberger Klimadialogs Ende April 2020 eine Allianz aus mehr als 60 Großunternehmen, darunter Thyssenkrupp, Allianz, Bayer, E.on, Puma und die Telekom (Stiftung 2° 2020). Ähnliche Forderungen formuliert das Beratungsgremium der Bundesregierung in Nachhaltigkeitsfragen, der Rat für Nachhaltige Entwicklung (RNE), in einem Positionspapier vom Mai 2020: „Für die Bewältigung der Pandemie-Folgen in Wirtschaft und Gesellschaft wird es entscheidend sein, dass die Weichen von Beginn an richtig im Sinne der globalen Nachhaltigkeitsziele gestellt werden“ (RNE 2020:1).

Während entsprechende, an Nachhaltigkeitskriterien ausgerichtete Klima-Konjunkturprogramme nicht nur innerhalb der EU geboten sind, um die Doppelkrise zu bewältigen und mit einem innovativen Wirtschaftsmodell gestärkt aus ihnen hervorzugehen, scheint die Anwendung eines solchen Ansatzes für die Mehrzahl der Schwellen- und Entwicklungsländer dringend notwendig: Die Auswirkungen der Krise im Globalen Süden sind aktuell – Mitte 2020 – kaum abzusehen. Zwar ist die dortige Bevölkerung im Durchschnitt häufig jünger und somit offenbar nicht so gefährdet. Dem stehen jedoch erheblich schlechter entwickelte Gesundheitssysteme und Infrastrukturen gegenüber. Sinkende Rohstoffeinnahmen und ausbleibende Zahlungen von Familienangehörigen im Ausland erschweren die Situation weiter. Zudem sind die Gesundheitssysteme oft in einem schlechten Zustand, und die Krankenhäuser sind nicht hinreichend mit Intensivbetten ausgestattet. Die Situation verlangt es, seitens der G20-Staaten, des Internationalen Währungsfonds (IWF), der Entwicklungsbanken – jeweils unterstützt von der EU und ihren Einzelregierungen – eine entsprechende Strategie zu entwickeln (Bals et al. 2020:5).

## *Status der Treibhausgas-Emissionen*

Ein hierfür prädestinierter Anknüpfungspunkt ist der im Europäischen Green Deal verankerte Leitsatz, durch die neue EU-Klima-Roadmap die Klimadiplomatie der EU zu stärken und wirkungsvoller zu machen. In diesem Zusammenhang ist zu klären, wie dieser neue Gesellschaftsvertrag und sein Wirtschaftsmodell mit einer Hebelwirkung den globalen Wandel hin zu einer möglichst weltweiten Klimaneutralität voranbringen könnte (Mathieu et al. 2020), insbesondere im Hinblick auf Klimaallianzen mit Afrika und China.

Laut Levin et al. 2019 und der Climate Watch Datenplattform des World Resources Institute sind die weltweiten Treibhausgas-Emissionen (THG) vom Jahr 2016 auf das

Jahr 2017 um weitere 1,1 Prozent auf 47,6 Gigatonnen (Gt) $CO_2$-Äquivalente angestiegen. In Bezug auf die globalen $CO_2$-Emissionen zeichnet sich ein ähnliches Bild ab: Nach einer leichten Seitwärtsentwicklung zwischen den Jahren 2014 und 2016 befindet sich der $CO_2$-Ausstoß seit 2017 wieder auf einem klaren Wachstumspfad. So stiegen die $CO_2$-Emissionen im Jahr 2017 um 1,6 Prozent, 2018 um 1,7 Prozent und erste Prognosen für 2019 sagen eine Zunahme um 0,6 Prozent voraus. Der Anteil Chinas (mit 9,3 Gigatonnen) betrug im Jahr 2017 28 Prozent und war damit mit Abstand größter $CO_2$-Emittent weltweit. Die damals noch 28 EU-Staaten (EU-28, inklusive dem Vereinigten Königreich von Großbritannien und Nordirland) kamen zum gleichen Zeitpunkt (mit 3,5 Gigatonnen) auf 10,6 Prozent und Afrika (mit 1,2 Gigatonnen) auf lediglich 3,6 Prozent der globalen $CO_2$-Emissionen.

Aufgrund des wachsenden Energiehungers baut China deutlich mehr Kraftwerkskapazitäten hinzu als alte abgeschaltet werden – insbesondere neue Kohlekraftwerke (KKW). Gleichzeitig wächst die Kohleförderung im Land nach wie vor an. Seinen Höchststand an KKW-Kapazität will China erst bei rund 1300 Gigawatt (GW) installierter Leistung etwa um das Jahr 2030 erreichen. Dies bedeutet, dass in den nächsten zehn Jahren nochmals rund 300 Gigawatt an KKW-Kapazität hinzukommen. Allein dieser Zubau entspricht der gesamten KKW-Kapazität der USA und Deutschlands zusammen. 2018 belief sich der Kohleverbrauch Chinas auf rund 3,8 Milliarden Tonnen (plus 3 Prozent gegenüber dem Vorjahr), was in etwa der Hälfte des weltweiten Verbrauchs entsprach (Dombrowski 2019:49 f).

Betrachtet man die THG-Emissionen jedoch pro Kopf, ergibt sich ein unterschiedliches Bild: Hier lagen im Jahr 2018 die chinesischen Pro-Kopf Emissionen an $CO_2$ mit 7 Tonnen nahezu gleichauf mit denen der EU-28 mit 6,7 Tonnen, während ein Mensch auf dem afrikanischen Kontinent im Durchschnitt lediglich 1,1 Tonnen an $CO_2$ im gleichen Jahr verbrauchte. Ein wiederum anderes Bild ergibt sich, wenn man die historisch akkumulierten $CO_2$-Emissionen in den Blick nimmt. Diese lagen zusammengerechnet bis einschließlich des Jahres 2017 für die EU-28 bei 353 Gigatonnen $CO_2$, was einem Anteil von 22 Prozent des global bis zu diesem Zeitraum emittierten $CO_2$ entsprach. China stieß bis Ende 2017 insgesamt 200 Gigatonnen $CO_2$ aus und erreichte damit einen globalen akkumulierten Anteil von 12,7 Prozent. Afrika kam hingegen nur auf 43 Gigatonnen $CO_2$ bzw. auf einen Anteil von nur 3 Prozent.

Diese Betrachtungsweise macht die globale Verantwortung der EU in Bezug auf die Reduzierung von Klimagasen deutlich. Afrika hingegen ist der Kontinent mit den niedrigsten Pro-Kopf-Emissionen, aber den höchsten Raten für Bevölkerungs- und Wirtschaftswachstum. Der globale Trend zur Urbanisierung ist in afrikanischen Städten besonders stark ausgeprägt. Aus diesen Gründen geht der World Energy Outlook davon aus, dass Afrikas steigender Erdölverbrauch bis zum Jahr 2040 stärker ausfallen wird als der Chinas und der afrikanische Kontinent somit ein deutliches Treibhausgaspotenzial birgt (Levin et al. 2019; World Resources Institute/Climate Watch 2020; IEA 2019; Ritchie et al. 2019; Global Carbon Atlas 2020).

## *European Green Deal – Europas „Mensch auf dem Mond“?*

Der IWF hat im World Economic Outlook von April 2020 ein negatives Wirtschaftswachstum von minus 3 Prozent für das Jahr 2020 angegeben. Damit wurde die Schätzung von Januar 2020 um weitere 6,8 Prozentpunkte reduziert. Die Wachstumsaussichten sind nun deutlich niedriger als zur Finanzkrise 2008/2009. Allerdings gehen die Ökonomen davon aus, dass sich die Weltwirtschaft wieder erholen kann, wenn nationale sowie internationale politische und monetäre Maßnahmen wirken (IMF 2020).

Hierzu kann der am 11. Dezember 2019 von der EU-Kommission vorgestellte European Green Deal einen wesentlichen Teil beitragen. Denn er ist das erste politische Großprojekt von Kommissionpräsidentin Ursula von der Leyen, die den Green Deal als Europas „Mann-auf-dem-Mond-Moment“ bezeichnete. Darin verankert ist das ehrgeizige Ziel, Europa bis zum Jahr 2050 zum ersten klimaneutralen Kontinent zu machen. Ein umfassender Maßnahmenkatalog sieht eine Umstrukturierung der Wirtschaft hin zu ökologischem Wachstum, eine Revision des EU-Emissionshandels, die Prüfung einer $CO_2$-Steuer für Importe, eine Neuausrichtung von Wertschöpfungsketten sowie Investitionen in grüne Technologien vor (Europäische Kommission 2020a).

Als ersten Schritt hat die Kommission am 4. März 2020 einen Entwurf für ein Europäisches Klimagesetz vorgelegt, das die Treibhausgasneutralität bis 2050 als rechtsverbindliches Ziel festschreibt. Demnach darf in der EU nur noch so viel $CO_2$ ausgestoßen werden, wie von der Natur wieder aufgenommen werden kann. Um dies zu erreichen, ist eine Neubewertung der Reduktionsvorgabe der EU für Treibhausgasemissionen bis 2030 vorgesehen. Auf der Grundlage einer umfassenden Folgenabschätzung und unter Berücksichtigung der integrierten nationalen Klima- und Energiepläne lotet die Kommission bis September 2020 die Option aus, die Emissionsreduktion auf 50 bis 55 Prozent gegenüber 1990 anzuheben und damit spürbar über das bisherige Reduktionsziel für 2030 in Höhe von 40 Prozent hinauszugehen (Europäische Kommission 2020b).

Weiterhin schlägt die Kommission vor, für den Zeitraum von 2030 bis 2050 einen EU-weiten Zielpfad für die Verringerung der Treibhausgasemissionen festzulegen. Dafür ist vorgesehen, bis September 2023 und danach alle fünf Jahre zu prüfen, ob die Maßnahmen der Mitgliedsstaaten mit dem Ziel der Klimaneutralität und dem Zielpfad im Einklang stehen.

Um die ambitionierten Zielsetzungen erreichen zu können, muss eine erhebliche Investitionslücke geschlossen werden. Die ursprüngliche Planung der Kommission

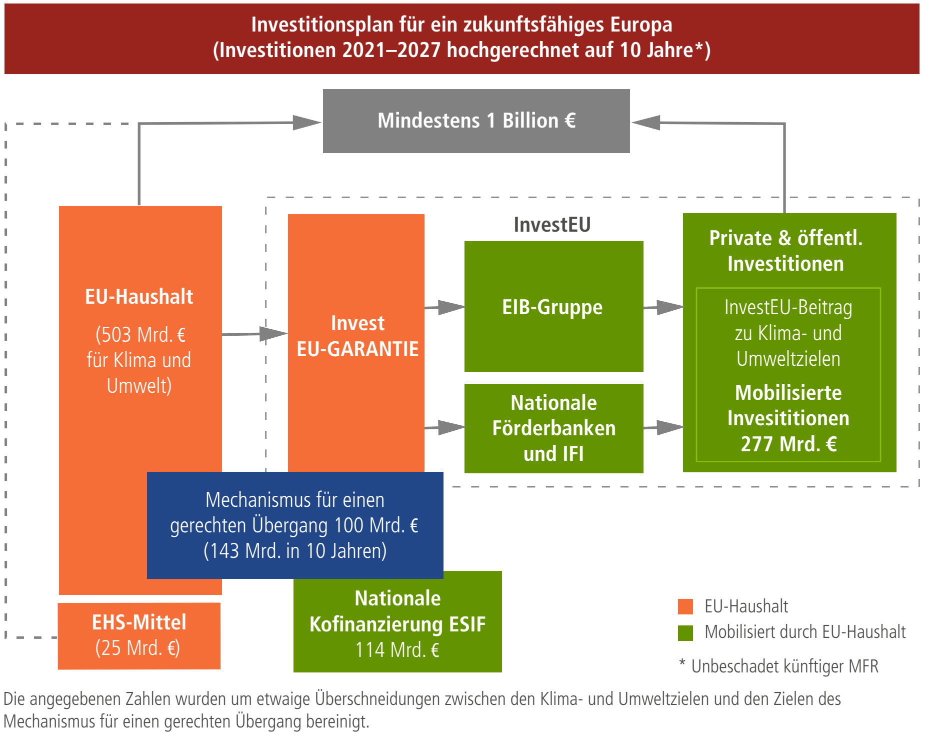

*Abb. 4.1 Investitionsplan für ein zukunftsfähiges Europa. (Investitionen 2021–2027 hochgerechnet auf 10 Jahre, eigene Darstellung nach Europäische Kommission 2020c)*

wurde im Januar 2020 in Form eines Investitionsplans für den europäischen Grünen Deal (IPEGD) vorgestellt (siehe Abb. 4.1). Dieser sieht Investitionen in Höhe von mindestens einer Billion Euro über die nächsten zehn Jahre vor. Werden alle Mittel zusammengenommen und von 7 auf 10 Jahre hochgerechnet, erreicht der Beitrag aus dem EU-Haushalt 503 Milliarden Euro, nationale Kofinanzierungen betragen 114 Milliarden Euro und private und öffentliche Klima- und Umweltinvestitionen über das Programm InvestEU rund 279 Milliarden Euro (Europäische Kommission 2020d). Allerdings sind die ursprünglich geplanten monetären Pläne des Green Deals vor dem Hintergrund der Corona-Pandemie keine Selbstverständlichkeit. Hohe finanzielle Mittel, die zur Bekämpfung der Corona-Ausbreitung und deren Folgen an die am stärksten betroffenen Staaten fließen, werden teilweise in der ursprünglichen Planung fehlen. Eine finale Festlegung finanzieller Maßnahmen bleibt deshalb abzuwarten. Allerdings dürfte schon jetzt klar sein, dass es nach der Pandemie nicht weitergeht wie zuvor. Tatsächlich sind zusätzliche Investitionen in saubere Technologien und Klimaschutz wahrscheinlich (TAZ 2020).

In direktem Bezug zum Green Deal steht die europäische Industriestrategie, die am 10. März 2020 veröffentlicht wurde. Sie stellt den Rahmen für die Umstrukturierung der Wirtschaft dar, sodass die europäische Industrie beim Übergang zur Klimaneutralität wettbewerbsfähig bleibt und europäische Firmen langfristig eine globale Führungsrolle bei der Digitalisierung einnehmen. Damit ist die Industriestrategie auch vor dem Hintergrund der Corona-Krise relevant, die verdeutlicht hat, dass in der EU Abhängigkeiten von außereuropäischen Lieferanten bestehen. Erklärtes Ziel der Industriestrategie ist es, die Größe und Integration des EU-Binnenmarktes als Hebel zu nutzen, um globale Standards zu setzen. Dadurch sollen europäische Werte verbreitet, strategische Autonomie gesichert und die Wettbewerbsfähigkeit der Industrie gestärkt werden (Europäische Kommission 2020e).

Der Green Deal und die Industriestrategie haben das Potenzial, den Wandel hin zu einer klimaneutralen Wirtschaft und Gesellschaft in Europa und über Wertschöpfungs- und Lieferketten weltweit anzustoßen. Weiterhin dürften europäische Zielvorgaben wie auch in der Vergangenheit Einfluss auf regulatorische Entscheidungen in anderen Staaten der Welt haben. Christoph Bals, politischer Geschäftsführer von Germanwatch, stellt fest: „Länder wie China und Indien brauchen eine frühe Zielankündigung der EU, um mit diesem Rückenwind ihre eigene Zielanhebung zu Hause und im Dialog mit der EU ausreichend diskutieren zu können" (Germanwatch 2020).

Gerade vor dem Hintergrund der Bewältigung der Corona-Pandemie bietet sich ein Handlungsfeld, um auf Schlüsselstaaten zuzugehen und unter Berücksichtigung der Klimaschutzziele sektorale Transformationen anzutreiben. Unter Berücksichtigung ethischer und Sicherheitsaspekte können in vielen Bereichen neue Kooperationsformate etwa mit China und afrikanischen Staaten initiiert werden. Denn das Kooperations- und Marktpotenzial in neuen und nachhaltigen Technologiefeldern ist enorm. Damit bieten der Green Deal und die Industriestrategie einen kraftvollen Hebel für Technologie-Kooperationen weltweit und können so zielgerichtet zur Verwirklichung der Klimaschutzziele beitragen.

## *China und Afrika zeigen, dass Handlungsbedarf besteht*

Die Corona-Krise hat verdeutlicht, wie eng verflochten China und die Welt mittlerweile sind. Denn die wirtschaftliche Entwicklung Chinas, verbunden mit zunehmend globalisierten Lieferketten und umfassendem Personenverkehr, haben die schnelle Ausbreitung des Virus innerhalb und aus China heraus begünstigt. Die Pandemie konnte sich so aus der Stadt Wuhan in der Provinz Hubei nahezu weltweit ausbreiten (Johns Hopkins University 2020).

Um Abhängigkeiten bei Lieferketten etwa von Schutzausrüstung wie Beatmungsgeräten und Gesichtsmasken aus China zu reduzieren, wurden in Deutschland und anderen Staaten Überlegungen laut, die Produktion in diesen Bereichen aus China abzuziehen bzw. zu verlagern. Die japanische Regierung hat sogar ein Finanzierungspaket von 220 Milliarden Yen (1,87 Milliarden Euro) geschnürt, um japanische Unternehmen dazu zu bewegen, Teile der Produktion aus China in andere Länder zu verlagern. Auch in den USA gibt es derartige Überlegungen (Reynolds et al. 2020).

Allerdings können über Jahrzehnte gewachsene Clusterstrukturen für Produktion und Zulieferer in China nicht ohne Weiteres ersetzt werden. Vielmehr gilt es, die wirtschaftliche und Klima-Kooperation mit China neu zu justieren. Der Europäische Green Deal, die EU-Industriestrategie und die Integration des EU-Binnenmarkts bieten hierbei die Möglichkeit, dass Europa gegenüber China mit einer gemeinsamen Stimme spricht. Die Maßnahmen können als Hebel dienen, die Wettbewerbsfähigkeit der europäischen Industrie zu stärken, auf nachhaltige und umweltfreundliche Wirtschafts- und Klimapolitik in China hinzuwirken und dabei die strategische Autonomie in Europa zu gewährleisten.

Dabei hat China durchaus ein offenes Ohr für neue Kooperationsformate mit der EU. Denn das Land befindet sich in einem langwierigen Handelskonflikt mit den USA und ist auf Partner aus Europa angewiesen. Zudem hat die Corona-Pandemie noch einmal die Dringlichkeit zu handeln verdeutlicht: China hat gravierende ökologische Probleme, resultierend aus der rasanten Urbanisierung, der Verschmutzung von Luft, Böden und Gewässern, der Wasserknappheit und Desertifikation im Norden sowie den zunehmenden Naturkatastrophen im Süden. Mit der Ratifizierung des Pariser Klimaschutzabkommens 2015 hat sich China erstmalig auf verbindliche Klimaschutzziele eingelassen und einen nationalen Emissionshandel beschlossen. Den strategischen Rahmen für Chinas Klimaziele bieten der 13. Fünfjahresplan (2016–2020) und seine Unterpläne, die eine Restrukturierung der Wirtschaft hin zu einem langsameren und nachhaltigeren und innovativeren Wachstum fokussieren. So plant das Land, den Kohleverbrauch bis 2020 von 64 Prozent auf 58 Prozent zu reduzieren sowie den Anteil der Erneuerbaren auf 68 Prozent der neu installierten Kapazitäten zu steigern (GWEC o.J.).

Bei Klima-Kooperationen mit China sollten die EU-Staaten in der EU geltende Umweltstandards und Standards beim Emissionshandel als gemeinsame Grundlage für Verhandlungen nutzen. Dabei kann die seit 2005 bestehende Partnerschaft im Bereich Klimawandel ein Ausgangspunkt sein. In diesem Rahmen wurde bereits von 2014 bis 2017 ein Projekt zur Konzeption und Verwirklichung des Emissionshandels in China durchgeführt. Das Projekt diente in seiner ersten Phase der Unterstützung der sieben chinesischen Pilotsysteme für die Etablierung eines nationalen Emissionshandelssystems. In einer zweiten Phase von 2017 bis 2020 unterstützt das EU-chinesische Projekt China bei der konkreten Umsetzung des nationalen Emissionshandelssystems, das 2017 offiziell aufgesetzt wurde (EU-China Emissions Trading System 2020).

Die Zusammenarbeit mit China ist auch auf dem Gebiet der Biodiversität vielversprechend. Im Green Deal wird konkret die Kooperation in diesem Bereich auch im Hinblick auf die 15. Vertragsstaatenkonferenz (COP15) der Biodiversitätskonventionen angesprochen, deren aktualisiertes Datum aufgrund der Corona-Pandemie allerdings noch nicht feststeht.

Darüber hinaus kann auf bestehende bilaterale Formate zwischen Deutschland, weiteren europäischen Ländern und China aufgebaut werden. So befindet sich die deutsch-chinesische Klimapartnerschaft im Rahmen der Internationalen Klimaschutz-Initiative (IKI) des Bundesumweltministeriums derzeit in der dritten Phase (BMU 2020). Zudem werden unter dem Rahmenprogramm Forschung für Nachhaltige Entwicklung (FONA) des Bundesforschungsministeriums (BMBF 2019) erfolgreich Projekte im Bereich „Sauberes Wasser" gefördert, und im Oktober 2019 wurde die Förderung von Projekten im Bereich der Klimaforschung initiiert.

Der EU Green Deal bietet die Voraussetzungen, Kooperationen mit China in diesen Politikfeldern auf die europäische Ebene zu heben und auf weitere Bereiche auszuweiten. Dabei ist entscheidend, dass die Modalitäten für den Wissens- und Technologieaustausch vorab geklärt werden. Bei Kooperationsformaten in China gilt dies vor allem für die Bereiche geistiges Eigentum, Datenschutz und die Wahl der Rechtsform wie etwa der Joint-Venture-Zwang oder die Einstufung als Nichtregierungsorganisation. Von Vorteil ist sicherlich, dass bei Kooperationen im Umwelt- und Klimabereich die Anwendung von Technologien in ethisch und moralisch umstrittenen Bereichen eher unwahrscheinlich ist.

Neben China bietet sich für die EU auch mit Afrika eine potenziell für beide Seiten gewinnbringende Möglichkeit, im Zeichen der für beide Kontinente noch kaum abzuschätzenden sozialen, wirtschaftlichen und ökologischen Folgeschäden der Corona-Pandemie, gemeinsame klima- und konjunkturpolitische Anstrengungen anzustoßen.

Eine entsprechende Kooperation auf Augenhöhe wäre dabei in mehrfacher Hinsicht sinnvoll: Zum einen aufgrund der soziodemografischen Entwicklung Afrikas und den damit verbundenen Herausforderungen Bevölkerungswachstum und Beschäftigung. Die Bevölkerung Afrikas hat sich in den vergangenen 30 Jahren auf 1,25 Milliarden verdoppelt und wird sich bis 2050 voraussichtlich noch einmal auf 2,5 Milliarden verdoppeln. Rund 800 Millionen Frauen und Männer werden zwischen 2020 und 2050 ins Erwerbsleben eintreten – im Durchschnitt 27 Millionen Arbeitsuchende pro Jahr. Afrika wird somit drei Viertel der Zunahme in der globalen Erwerbsbevölkerung in diesen 30 Jahren ausmachen (Arnold 2019). Um diese Entwicklung aufzufangen, bedarf es einer Transformation der afrikanischen Wirtschaft hin zu einer nachhaltigen Entwicklung. Europa ist aufgrund der enormen ökonomischen Bedeutung für Afrika prädestiniert, eine solche ökologische Transformation partnerschaftlich zu begleiten, denn die EU ist Afrikas größer Handels- und Investitionspartner und mit einem Beitrag von 72,5 Millionen Euro bis Ende 2020 auch wichtigster Unterstützer der Afrikanischen kontinentalen Freihandelszone (AfCFTA). Im Jahr 2018 erreichte der Warenhandel zwischen den 27 EU-Mitgliedstaaten und Afrika einen Gesamtwert von 235 Milliarden Euro – dies entspricht 32 Prozent des gesamten Handelsvolumens von Afrika. Im Vergleich dazu erreichte der Handel zwischen Afrika und China einen Wert von 125 Milliarden Euro (17 Prozent) und mit den USA 46 Milliarden Euro (6 Prozent). Im Jahr 2017 belief sich der Bestand der ausländischen Direktinvestitionen der 27 EU-Mitgliedstaaten in Afrika auf 222 Milliarden Euro – mehr als das Fünffache der Investitionen der USA (42 Milliarden Euro) oder Chinas (38 Milliarden Euro) (Europäische Kommission 2020 f: 3).

Andererseits kann die EU mit einem entsprechenden Wissens- und Technologietransfer zur Entwicklung der afrikanischen Länder beitragen und gleichzeitig gewährleisten, dass die im Rahmen des EU Green Deal für Europa gesteckten Nachhaltigkeitsstandards, ähnlich wie in einer entsprechenden Kooperation mit China, auch in Afrika zum Tragen kommen und somit auch auf internationaler Ebene ihre Wirkung entfalten können.

Ein erster Schritt in diese Richtung ist die im März 2020 im Zeichen des EU Green Deals vorgestellte Mitteilung der EU-Kommission „Auf dem Weg zu einer umfassenden Strategie mit Afrika“. Darin werden neue Wege der Zusammenarbeit vorgeschlagen, um die strategische Allianz der EU mit Afrika zu stärken und die wachsende Zahl an globalen Herausforderungen gemeinsam anzugehen, wobei die Auswirkungen des Klimawandels und der digitale Wandel an erster Stelle genannt werden. Diese beiden Themen sollen auch Schwerpunkte einer künftigen intensivierten Kooperation sein. Neben der grünen Wende, dem Energiezugang und dem digitalen Wandel wurden nachhaltiges Wachstum und nachhaltige Beschäftigung, Frieden und Governance sowie Migration und Mobilität als weitere zentrale Kooperationsfelder festgelegt.

Für jeden dieser Themenbereiche wird im Strategievorschlag dargelegt, wie die gemeinsamen Ziele erreicht werden können. Zudem wird betont, dass der Aufbau einer starken politischen und vertieften Zusammenarbeit in globalen und multilateralen Angelegenheiten im Mittelpunkt des gemeinsamen Handelns stehen soll.

Um diese Ziele zu verwirklichen, setzt sich die EU in der Partnerschaft mit Afrika für eine Maximierung des Nutzens der grünen Wende ein, wobei die Prioritäten gleichlautend wie im EU Green Deal gesetzt sind: Kreislaufwirtschaft, nachhaltige Wertschöpfungsketten und Lebensmittelsysteme, die Förderung erneuerbarer Energien, die Verringerung von Emissionen, der Schutz der biologischen Vielfalt und der Ökosysteme bis hin zur Förderung grüner und nachhaltiger Urbanisierungsmodelle (Europäische Kommission 2020 f).

Um die grüne Wende zu realisieren, setzt die EU auf Innovationen. Deshalb sollten notwendige Investitionen in nachhaltige Entwicklung darauf ausgerichtet sein, die wissenschaftlichen Kapazitäten in Afrika zu stärken, indem sie den Zugang zu und die lokale Anpassung an Technologien ermöglichen. Dies wird die afrikanischen Länder in die Lage versetzen, in einen kohlenstoffarmen, klimaresistenten und grünen Wachstumspfad zu investieren, der ineffiziente Technologien wie etwa Kohleverstromung vermeidet und stattdessen erneuerbare Energiequellen erschließt.

Um eine entsprechende grüne Wende in Afrika anzustoßen, empfiehlt die EU-Kommission insbesondere folgende Maßnahmen:

- Unterstützung bei der Umsetzung nationaler Klimaschutzziele (Englisch: nationally determinded contributions, NDCs) im Rahmen des Pariser Klimaschutzabkommens, bei der Ausarbeitung ehrgeiziger langfristiger Strategien zur Verringerung der THG-Emissionen sowie bei der Entwicklung von nationalen Anpassungsplänen.
- Einleitung einer „Green Energy“-Initiative, aufbauend auf den Empfehlungen der „High Level Platform for Sustainable Energy Investments in Africa“ (SEI Platform) und der Allianz Afrika-Europa für nachhaltige Investitionen und Arbeitsplätze (Europäische Kommission 2020g).

## *Ausblick*

Wie sich an den Beispielen China und Afrika zeigt, weist der EU Green Deal das Potenzial auf, Katalysator für neue Klimaallianzen und damit in erweiterter Konsequenz auch für eine Wiederbelebung der internationalen Klimadiplomatie zu sein. Hinsichtlich der notwendigen Verzahnung mit den Post-Corona-Konjunkturprogrammen stellen sich zwei grundlegende Fragen für die nähere Zukunft. Was sind die notwendigen nächsten Schritte

- in Bezug auf eine möglichst konsequente Umsetzung des EU Green Deals, vor allem in Bezug zur EU-Industrie-Strategie und zum Aktionsplan für eine Kreislaufwirtschaft (siehe Kap. 13 „Kreislaufwirtschaft als Säule des EU Green Deal“ dieses Buches)
- für die Realisierung der angestrebten Klimaallianzen als Mechanismen zur Hebung der Synergien zur Bewältigung der Folgen der Corona-Krise und des Klimawandels?

Diesen beiden Fragestellungen übergeordnet stellt sich zudem die Frage, wie eine möglichst auf einer nachhaltigen Entwicklung basierende Verknüpfung von geplanten Aktivitäten innerhalb des EU Green Deals und der Corona-Konjunkturpakete tatsächlich gewährleistet werden kann.

Zunächst wäre sicherzustellen, dass Corona-Hilfspakete der EU möglichst an klimarelevante Bedingungen geknüpft werden. Bis Ende April 2020 wurden allein seitens der EU-Mitgliedsstaaten bis zu 1,8 Billionen Euro an nationalen Konjunkturprogrammen aufgelegt, die die EU-Kommission durchweg genehmigte. Weiterhin hat die EU selbst Anfang April 2020 zunächst 540 Milliarden Euro zur direkten Bekämpfung der coronabedingten wirtschaftlichen Schäden bereitgestellt. Auch die Europäische Zentralbank plant Anleihenkäufe für konjunkturbedingte Maßnahmen im Umfang von bis zu 870 Milliarden Euro, wobei diese nicht explizit nach klimafreundlichen Kriterien vergeben werden (Schulz 2020).

Umso wichtiger erscheint die Berücksichtigung derartiger Klimaschutz-Vergaberegeln für die spätere Erholungsphase, wie etwa die Technische Expertengruppe (TEG) der EU-Kommission zum Thema nachhaltige Finanzen in einer Ende April 2020 veröffentlichten Stellungnahme dringend empfiehlt. Insbesondere gilt dies für den von der EU-Kommission geplanten bis zu 1000 Milliarden Euro schweren Post-Corona-Wiederaufbauplan (Englisch: Roadmap to Recovery). Die TEG fordert, dass die Transformation hin zu einer klimaneutralen Wirtschaft zu einer Grundlage der Wiederaufbaupläne gemacht werden sollte. Eines der entscheidenden Vergabekriterien könnte dabei die grüne Taxonomie werden, auf die sich der Rat der Europäischen Union und das EU-Parlament bereits im Dezember 2019 geeinigt hatten. Bei der grünen Taxonomie handelt es sich um ein europaweit einheitliches Klassifikationssystem für nachhaltige Wirtschaftstätigkeiten. Die Taxonomie soll die Fragmentierung des Marktes an Nachhaltigkeitslabeln im Finanzbereich beseitigen und sogenanntes Greenwashing verhindern, bei dem gegenüber Anlegern Finanzprodukte als grüner – also umweltfreundlicher – beworben werden, als sie es nachweislich sind (ZIA 2020). Rechtlich bindend soll diese Taxonomie eigentlich erst Ende des Jahres 2021 sein.

Die TEG empfiehlt nun eine Integration der grünen Taxonomie bereits in den EU-Wiederaufbauplan ebenso wie die Anwendung der schon entwickelten TEG-Standards für grüne Anleihen (Englisch: Green Bonds Standards) und den Vergleichsmaßstab für den EU-Klimawandel anhand der Beschlüsse des Pariser Klimaabkommens als Maßstab (Englisch: Paris-Aligned and Climate Transition Benchmarks) (TEG 2020; Schulz 2020). Unterstützung erhält die TEG-Gruppe durch eine neue Studie der Universität Oxford. Deren wichtigste Schlussfolgerung: Mit klimafreundlichen Konjunkturprogrammen können Staaten die Wirtschaft nach der Corona-Krise wirksamer und langfristiger stärken als mit Programmen ohne Fokus auf Klimaschutz. Demnach schaffen grüne Anschubhilfen im Bereich erneuerbare Energien mehr Arbeitsplätze, liefern kurzfristig höhere Kapitalrenditen und führen zu höheren langfristigen Kostensenkungen als herkömmliche fiskalische Anreizprogramme. Geeignete Investitionsfelder sind Wind- und Solarenergie, energetische Gebäudesanierung, Forschung und Entwicklung (FuE) bei grünen Technologien, Bildung an sich sowie Ausbildung und – vor allem in Entwicklungsländern – nachhaltige Landwirtschaft (Schaudwet 2020a; Hepburn et al. 2020).

Wichtig wird sein, dass sich die aufgezeigten Instrumente für die Vergabe von Staatshilfen und die im EU Green Deal und der EU-Industriestrategie genannten prioritären Aktionsfelder und -instrumente nicht nur für die EU-Mitgliedsstaaten als neue Handlungsmaxime hinsichtlich der notwendigen Verzahnung der Bekämpfung der Klima- und der Corona-Krise herausbilden. Vielmehr sollten sie auf China, Afrika und weitere an einer nachhaltigen Klimapolitik in einer Post-Corona-Ära interessierte Staaten ausgedehnt werden.

Ein weiterer Ansatzpunkt zur Kooperation ist die konzertierte Nutzung der im EU Green Deal gegebenen Möglichkeit des $CO_2$-Grenzausgleichsmechanismus (Englisch: carbon border adjustment mechanism), mit dem ab dem Jahr 2021 Importe aus ausgewählten Branchen außerhalb der EU mit einem quasi $CO_2$-Preis belegt werden können, um das Risiko von sogenannter Kohlenstoffleckage (Englisch: carbon leakage), also der Abwanderung $CO_2$-intensiver Produktion in Länder mit weniger strikten Emissionsregularien, zu reduzieren. Verbunden mit dem durch die Corona-Pandemie gestärkten Bewusstsein für Produktionssicherheit am eigenen Standort könnte dieser Mechanismus einen Hebel bieten, internationale Klima-Kooperationen zum Einsatz von mehr erneuerbaren Energien voranzutreiben.

Zudem stellt auch der Rat für Nachhaltige Entwicklung fest, dass international transparente, an ökologischen und sozialen Standards ausgerichtete Lieferketten widerstandsfähiger gegen Krisen sind und die wirtschaftlichen Perspektiven von Schwellen- und Entwicklungsländern verbessern können (RNE 2020; Schaudwet 2020b). Sollte dies gelingen, könnte der EU Green Deal tatsächlich als ein neuer, nicht auf der Ausbeutung von Mensch und Umwelt basierender Gesellschaftsvertrag eine spürbare Hebelwirkung im globalen Wandel hin zur Klimaneutralität entfalten (Bals et al. 2020; Mathieu 2020).

## References

[CR1] Arnold, Tom (2019): „Ländliches Afrika“: Die Partnerschaft mit Europa braucht Impulse. Online verfügbar unter www.welthungerhilfe.de/welternaehrung/rubriken/agrar-ernaehrungspolitik/eu-partnerschaft-fuer-das-laendliche-afrika/, zuletzt geprüft am 12.5.2020.

[CR2] Bals, Christoph; Mathieu, Audrey; Herzig, Linus (2020): Positionspapier – Corona- und Klimakrise: Europäischen Green Deal zur Bekämpfung der Doppelkrise nutzen. Online verfügbar unter www.germanwatch.org/de/18568, zuletzt geprüft am 11.5.2020.

[CR3] Bundesministerium für Bildung und Forschung (BMBF) (2019): Deutsch-Chinesisches „Forschungs- und Innovationsprogramm Sauberes Wasser“. Online verfügbar unter www.fona.de/de/massnahmen/internationales/deutsch-chinesisches-forschungs-und-innovationsprogramm-sauberes-wasser.php, zuletzt geprüft am 13.5.2020.

[CR4] Bundesministerium für Umwelt, Naturschutz und nukleare Sicherheit (BMU) (2020): Deutsch-chinesische Klimapartnerschaft. Online verfügbar unter www.international-climate-initiative.com/de/details/project/deutschchinesische-klimapartnerschaft-11_I_137-165, zuletzt geprüft am 13.5.2020.

[CR5] Carbon Tracker (2020): Political decisions, economic realities: The underlying operating cashflows of coal power during COVID-19. Online verfügbar unter https://carbontracker.org/reports/political-decisions-economic-realities, zuletzt geprüft am 12.5.2020.

[CR6] Dombrowski K (2019). Zu viele schwarze Schafe. Neue Energie.

[CR7] EU-China Emissions Trading System (2020). Online verfügbar unter www.eu-chinaets.org/, zuletzt geprüft am 13.5.2020.

[CR8] Europäische Kommission (2020a): European Green Deal. Online verfügbar unter https://eur-lex.europa.eu/legal-content/EN/TXT/?qid=1588580774040&uri=CELEX, zuletzt geprüft am 13.05.2020.

[CR9] Europäische Kommission (2020b): Europäisches Klimagesetz, Vorschlag für eine Verordnung. Online verfügbar unter https://ec.europa.eu/info/law/better-regulation/have-your-say/initiatives/12108-Climate-Law, zuletzt geprüft am 13.5.2020.

[CR10] Europäische Kommission (2020c): Mitteilung der Kommission an das Europäische Parlament, den Rat, den Europäischen Wirtschafts- und Sozialausschuss und den Ausschuss der Regionen – Investitionsplan für ein zukunftsfähiges Europa – Investitionsplan für den europäischen Grünen Deal. Online verfügbar unter https://ec.europa.eu/commission/presscorner/api/files/attachment/860464/Commission%20Communication%20on%20the%20European%20Green%20Deal%20Investment%20Plan_DE.pdf.pdf, zuletzt geprüft am 11.5.2020.

[CR11] Europäische Kommission (2020d): Investitionsplan für den europäischen Grünen Deal (IPEGD). Online verfügbar unter https://ec.europa.eu/commission/presscorner/detail/de/qanda_20_24, zuletzt geprüft am 13.5.2020.

[CR12] Europäische Kommission (2020e): Europäische Industriestrategie. Online verfügbar unter https://ec.europa.eu/info/strategy/priorities-2019-2024/europe-fit-digital-age/european-industrial-strategy_de, zuletzt geprüft am 13.5.2020.

[CR13] Europäische Kommission (2020f): Fragen und Antworten: Auf dem Weg zu einer umfassenden Strategie mit Afrika. Online verfügbar unter https://ec.europa.eu/commission/presscorner/api/files/document/print/de/qanda_20_375/QANDA_20_375_DE.pdf, zuletzt geprüft am 12.5.2020.

[CR14] Europäische Kommission (2020g): Joint Communication to the European Parliament and the Council – Towards a comprehensive strategy with Africa – Join(2020) 4 final. Online verfügbar unter https://ec.europa.eu/international-partnerships/system/files/communication-eu-africa-strategy-join-2020-4-final_en.pdf, zuletzt geprüft am 12.5.2020.

[CR15] Germanwatch (2020): EU-Klimagesetz: Entwurf ist ein Meilenstein mit Nachbesserungsbedarf. Online verfügbar unter https://germanwatch.org/de/18346, zuletzt geprüft am 13.5.2020.

[CR16] Global Carbon Atlas (2020): CO_2_ Emissions. Online verfügbar unter www.globalcarbonatlas.org/en/CO2-emissions, zuletzt geprüft am 02.6.2020.

[CR17] Global Wind Energy Council (GWEC) (o.J.): China’s new Five-Year Energy Plan. Online verfügbar unter https://gwec.net/chinas-new-five-year-energy-plan/, zuletzt geprüft am 13.5.2020.

[CR18] Hepburn, Cameron, O’Callaghan, Brian; Stern, Nicholas; Stiglitz, Joseph; Zenghelis, Dimitri (2020): Will COVID-19 fiscal recovery packages accelerate or retard progress on climate change?, Smith School Working Paper 20-02. Online verfügbar unter www.smithschool.ox.ac.uk/publications/wpapers/workingpaper20-02.pdf, zuletzt geprüft am 12.5.2020. 10.1093/oxrep/graa015 PMC7239121 40504141

[CR19] International Energy Agency (IEA) (2019): World Energy Outlook 2019 – Executive Summary. Online verfügbar unter https://iea.blob.core.windows.net/assets/1f6bf453-3317-4799-ae7b-9cc6429c81d8/English-WEO-2019-ES.pdf, zuletzt geprüft am 12.5.2020.

[CR20] International Monetary Fund (IMF): Word Economic Outlook April 2020. Online verfügbar unter www.imf.org/en/Publications/WEO/Issues/2020/04/14/weo-april-2020, zuletzt geprüft am 13.5.2020.

[CR21] Johns Hopkins University (2020): COVID-19 Dashboard. Online verfügbar unter https://gisanddata.maps.arcgis.com/apps/opsdashboard/index.html, zuletzt geprüft am 13.5.2020.

[CR22] Klimareporter (2020): „Der Klimadiplomatie schadet die Verschiebung vielleicht nicht, dem Klima schon“. Online verfügbar unter www.klimareporter.de/klimakonferenzen/der-klimadiplomatie-schadet-die-verschiebung-vielleicht-nicht-dem-klima-schon, zuletzt geprüft am 11.5.2020.

[CR23] Levin, Kelly; Lebling, Katie (2019): CO2 Emissions Climb to an All-Time High (Again) in 2019: 6 Takeaways from the Latest Climate Data. Online verfügbar unter www.wri.org/blog/2019/12/co2-emissions-climb-all-time-high-again-2019-6-takeaways-latest-climate-data, zuletzt geprüft am 12.5.2020.

[CR24] Mathieu, Audrey; Bals, Christoph (2020): Der Europäische Green Deal: Erneuerungsprojekt als Chance für Klima und Mensch. Online verfügbar unter www.germanwatch.org/de/18336, zuletzt geprüft am 11.5.2020.

[CR25] Rat für Nachhaltige Entwicklung (RNE) (2020): Raus aus der Corona-Krise im Zeichen der Nachhaltigkeit. Online verfügbar unter www.nachhaltigkeitsrat.de/wp-content/uploads/2020/05/20200513_RNE_Empfehlung_Raus_aus_der_Krise_im_Zeichen_der_Nachhaltigkeit.pdf, zuletzt geprüft am 25.5.2020.

[CR26] Reynolds, Isabel; Urabe, Emi (2020): Japan to Fund Firms to Shift Production Out of China. Online verfügbar unter www.bloomberg.com/news/articles/2020-04-08/japan-to-fund-firms-to-shift-production-out-of-china, zuletzt geprüft am 13.5.2020.

[CR27] Ritchie, Hannah; Roser Max (2019): CO_2_ and Greenhouse Gas Emissions. Veröffentlicht auf OurWorldInData.org. Online verfügbar unter https://ourworldindata.org/co2-and-other-greenhouse-gas-emissions, zuletzt geprüft am 12.5.2020.

[CR28] Schaudwet, Christian (2020a): Grüne Hilfsprogramme helfen Wirtschaft mehr. Online verfügbar unter https://background.tagesspiegel.de/energie-klima/gruene-hilfsprogramme-helfen-wirtschaft-mehr, zuletzt geprüft am 12.5.2020.

[CR29] Schaudwet, Christian (2020b): RNE: Klimaschutz aufweichen schadet Wirtschaft. Online verfügbar unter https://background.tagesspiegel.de/energie-klima/rne-klimaschutz-aufweichen-schadet-wirtschaft, zuletzt geprüft am 25.5.2020.

[CR30] Schulz, Florence (2020): Wie die Coronahilfen der EU grün werden sollen. Online verfügbar unter https://background.tagesspiegel.de/energie-klima/wie-die-coronahilfen-der-eu-gruen-werden-sollen, zuletzt geprüft am 12.5.2020.

[CR31] Stiftung 2 ° (2020): Mit Klima-Konjunkturprogramm Wirtschaft krisenfester machen –Unternehmen senden starkes Signal vor Petersberger Klimadialog. Online verfügbar unter www.stiftung2grad.de/wp-content/uploads/2020/04/PM-2%C2%B0-Klimakonjunkturprogramm-3.pdf, zuletzt geprüft am 11.5.2020.

[CR32] TAZ (2020): EU bremst Green Deal. https://taz.de/Langsamer-Klimaschutz/, zuletzt geprüft am 13.5.2020.

[CR33] (EU) Technical Expert Group on Sustainable Finance (TEG) (2020a): Sustainable recovery from the Covid-19 pandemic requires the right tools. Online verfügbar unter https://ec.europa.eu/info/sites/info/files/business_economy_euro/banking_and_finance/documents/200426-sustainable-finance-teg-statement-recovery_en.pdf, zuletzt geprüft am 12.5.2020.

[CR34] World Resources Institute (2020): Climate Watch. Online verfügbar unter www.wri.org/our-work/project/climate-watch bzw. www.climatewatchdata.org, zuletzt geprüft am 12.5.2020.

[CR35] Zentraler Immobilien Ausschuss (ZIA) (2020): Sustainable Finance. Online verfügbar unter www.zia-deutschland.de/themen/sustainable-finance/, zuletzt geprüft am 12.5.2020.

